# Endodontic Microbiology—A Special Issue of Dentistry Journal

**DOI:** 10.3390/dj6020014

**Published:** 2018-05-17

**Authors:** Prasanna Neelakantan

**Affiliations:** Discipline of Endodontology, Faculty of Dentistry, The University of Hong Kong, Pok Fu Lam, Hong Kong, China; prasanna@hku.hk

**Keywords:** biofilm, EPS matrix, intracanal medicaments, endotoxin/lipopolysaccharide, lipoteichoic acid, nanoparticles, persistent infection, quorum sensing, root canal disinfection, root canal irrigants, reinfection, virulence

Understanding microbiology, specifically biofilm biology is an essential component of creating targeted therapeutic modalities that are effective and efficient. We have come to understand that pulpal and periradicular infections are biofilm mediated, and that this biofilm affords extraordinary resistance to microbial flora. This is due to biofilm structure, as well as the dynamics of the interactions between the microbial components of a biofilm. While there has been significant advancement in our understanding of microbial biofilms and their role in pulpal and periradicular diseases, much is still a mystery. The launch of this Special Issue, dedicated to “Endodontic Microbiology”, is thus timely, and aims to bring our readers exemplary work on this subject matter, both from a basic science perspective and from a translational perspective. We invite researchers to submit papers on this subject, which will enhance our understanding of endodontic microbiology.

